# Chimeric proteins tagged with specific 3xHA cassettes may present instability and functional problems

**DOI:** 10.1371/journal.pone.0183067

**Published:** 2017-08-11

**Authors:** Sara Saiz-Baggetto, Ester Méndez, Inma Quilis, J. Carlos Igual, M. Carmen Bañó

**Affiliations:** Departament de Bioquímica i Biologia Molecular and Estructura de Recerca Interdisciplinar en Biotecnologia i Biomedicina, Universitat de València, Burjassot (Valencia), Spain; National Taiwan University, TAIWAN

## Abstract

Epitope-tagging of proteins has become a widespread technique for the analysis of protein function, protein interactions and protein localization among others. Tagging of genes by chromosomal integration of PCR amplified cassettes is a widely used and fast method to label proteins *in vivo*. Different systems have been developed during years in the yeast *Saccharomyces cerevisiae*. In the present study, we analysed systematically a set of yeast proteins that were fused to different tags. Analysis of the tagged proteins revealed an unexpected general effect on protein level when some specific tagging module was used. This was due in all cases to a destabilization of the proteins and caused a reduced protein activity in the cell that was only apparent in particular conditions. Therefore, an extremely cautious approach is required when using this strategy.

## Introduction

Immunological methods have been revealed as crucial in the development of molecular and cellular biology. The use of antibodies recognising specific proteins has allowed researchers to investigate multiple protein aspects. However, obtaining antibodies against a protein of interest is a time and/or money consuming process and commercial antibodies against most of the proteins are not available or do not work properly. Because of that, fusion-tag technology has grown-up to facilitate protein analysis, purification and visualization. Nowadays, numerous types of tags with different features suited to diverse applications are available [1–4].

Fusion tags relevant for this manuscript can vary greatly in size, from large tags such as the *Aequorea victoria* green fluorescent protein (GFP) with a size of 26,9 kDa, to small peptide tags including multiple copies of c-myc and HA, each around 1 kDa. GFP tag is an invaluable tool for localizing proteins in both living and fixed cells [5–7], and both the HA epitope derived from influenza virus hemagglutinin [8], and the myc epitope derived from the mammalian c-myc protein [9], have been widely used for protein localization by immunofluorescence and for biochemical detection and isolation of proteins.

Different strategies for labelling proteins have been described over the years. A further breakthrough was achieved with the appearance of recombinant-based cloning vectors, which have made it unnecessary to create a new construct each time fusion to a new tag is desired, and even more, when genomic tagging was developed [10–12]. Very useful collections of sets of modules, which serve as templates for the PCR synthesis of fragments that allow a variety of gene modifications, have been developed for distinct model organisms. Among them, one that is widely used in the budding yeast *Saccharomyces cerevisiae* is the plasmid collection described in *Longtine et al*. [13]. These plasmids allow gene deletion, gene overexpression (using the regulable *GAL1* promoter), C- or N-terminal protein tagging (with GFP, GST, 3xHA or 13xmyc tags), and partial N- or C-terminal deletions (with or without concomitant protein tagging). Because of the modular nature of the plasmids, they allow an efficient and economical use of a small number of PCR primers for a wide variety of gene manipulations. Although new PCR-based strategy to generate yeast strains expressing endogenous levels of amino-terminal epitope–tagged proteins have been published [14], the widely used modules for N-terminal tagging rely upon the use of heterologous promoters; because of that, C-terminal tagged is often preferred as a first option, so this study focused on it.

It is frequently assumed that the protein tags used in biochemical experiments minimally perturb their host protein. However, it is necessary to be aware that this strategy could indeed involve risks since fusion proteins with small tags could have affected its tertiary structure, native function, stability or ability to interact with other proteins depending on the location and on the amino acids composition of the tag [15–20]. Serendipity, we have detected that this risk could be more extended than it was initially considered. In this report we carry out a systematic analysis in yeast of several proteins tagged with commonly used epitopes. Significant differences in protein expression caused by altered stability, impinging in cellular function, have been founded. Our results bring the risks of protein labelling techniques into the spotlight.

## Material and methods

### Yeast strains and growth conditions

The yeast strains used in this study are shown in Table 1. The 3xHA, myc and GFP tagging cassettes were amplified from pFA6a plasmid series [13] and integrated in the indicated parental strain. Three pFA6a-3HA plasmids obtained from different sources (including Addgene) were tested. The 6xHA tagging cassette was amplified from pGA2256 plasmid (a gift from Dr. G. Ammerer) and integrated in the indicated parental strain. The 3xHA^ΔIF^ cassette was amplified from pFA6a-3HA plasmid [13] using a forward primer lacking the codons for IF. An alternative 3xHA-*TRP1* cassette used for tagging Whi7 protein was obtained from Dr. M. Aldea [21].

**Table 1:** Yeast strains.

W303-1a	*MATa ade2-1 trp1-1 leu2-3*,*112 his3-11*,*15 ura3-52 can1 rad5-535*
YTR111^a^	*MATa his3Δ1 leu2Δ0 met15Δ0 ura3Δ0 ddc1*::*kanMX4*
JCY275	*cln1*::*kanMX6* in W303-1a
JCY411	*SLT2-3xHA-kanMX6* in W303-1a
JCY486	*SWI5-3xHA-TRP1* in W303-1a
JCY847	*cln1*::*kanMX6 cln2*::*TRP1* in W303-1a
JCY1357	*CLN2-3xHA-TRP1* in W303-1a
JCY1511	*PKC1-GFP-kanMX6* in W303-1a
JCY1544	*WHI7-3xHA-kanMX6* in W303-1a
JCY1661	*DDC1-GFP-kanMX6* in W303-1a
JCY1701	*DDC1-3xHA-kanMX6* in W303-1a
JCY1728	*WHI7-3xHA-TRP1* in W303-1a
JCY1825	*DDC1-6xHA-HIS3* in W303-1a
JCY1830	*CLN2-6xHA-HIS3* in W303-1a
JCY1887	*DDC1-myc-TRP1* in W303-1a
JCY1888	*CLN2-GFP-kanMX6* in W303-1a
JCY1890	*CLN2-myc-TRP1* in W303-1a
JCY1891	*PKC1-6xHA-HIS3* in W303-1a
JCY1901	*RAD53-6xHA-HIS3* in W303-1a
JCY1903	*RAD53-GFP-kanMX6* in W303-1a
JCY1905	*RAD53-3xHA-kanMX6* in W303-1a
JCY1907	*RAD53-myc-TRP1* in W303-1a
JCY1916	*PKC1-myc-TRP1* in W303-1a
JCY1929	*cln1*::*kanMX6* in JCY1357
JCY1960	*cln1*::*kanMX6* in JCY1830
JCY2033	*PKC1-3xHA-kanMX6* in W303-1a
JCY2063	*DDC1-3xHA*^*ΔIF*^*-kanMX6* in W303-1a

^a^from Dr. J.Torres

Cells were grown on standard yeast extract-peptone-dextrose (YPD) or synthetic dextrose (SD) medium supplemented with required amino acids. For growth assays, 10-fold serial dilutions were prepared from exponentially growing cultures (usually 2–8 × 10^6^ cells/mL) of the different strains. 5 μL of each dilution was then spotted onto YPD, YPD supplemented with 200 mM hydroxyurea (HU) or YPD followed by UV irradiation (40 J/m^2^) using the GS Gene Linker™ UV chamber (Bio-Rad).

### Stability assays and western blot

To evaluate protein stability translational shut-off experiments were carried out adding 100 μg/mL cycloheximide to exponentially growing cells. Samples were harvested at the indicated times and protein decay was analysed by western blot. Approximately 10^8^ cells were collected, resuspended in 100 μL water and, after adding 100 μL 0.2 M NaOH, they were incubated for 5 minutes at room temperature. Cells were collected by centrifugation, resuspended in 50 μL sample buffer and incubated for 5 minutes at 95°C. Extracts were clarified by centrifugation, and equivalent amounts of protein were resolved in an SDS-PAGE gel and transferred onto a nitrocellulose membrane. The primary antibodies used in this study include anti-Ddc1 (kindly provided by Dr. M. Muzi-Falconi), anti-Cln2 y-115 (Santa Cruz Biotechnology Inc.), anti-yeast Pkc1 (yC-20) (Santa Cruz Biotech Inc.), anti-Mpk1 (Slt2)-yC20 (Santa Cruz Biotechnology Inc.), anti-Rad53-YC19 (Santa Cruz Biotech Inc.), anti-Swi5 (kindly provided by Dr. D. Stillman), monoclonal anti-HA peroxidase 3F10 antibody (Roche Diagnostics), monoclonal anti-c-myc 9E10 antibody (Roche Diagnostics), monoclonal anti-GFP (Roche Diagnostics) and monoclonal anti Cdc2 p34 (PSTAIRE) sc-53 (Santa Cruz Biotech Inc.).

### Cell size analysis

Cell size was analysed in exponentially growing cultures after brief sonication in a Particle Count and Size Analyzer Z2 (Coulter). Graphs are the mobile average of histograms derived from at least five independent cultures.

## Results and discussion

### Protein level is affected by C-terminal tagging with a commonly used 3xHA epitope

**Fig 1:**
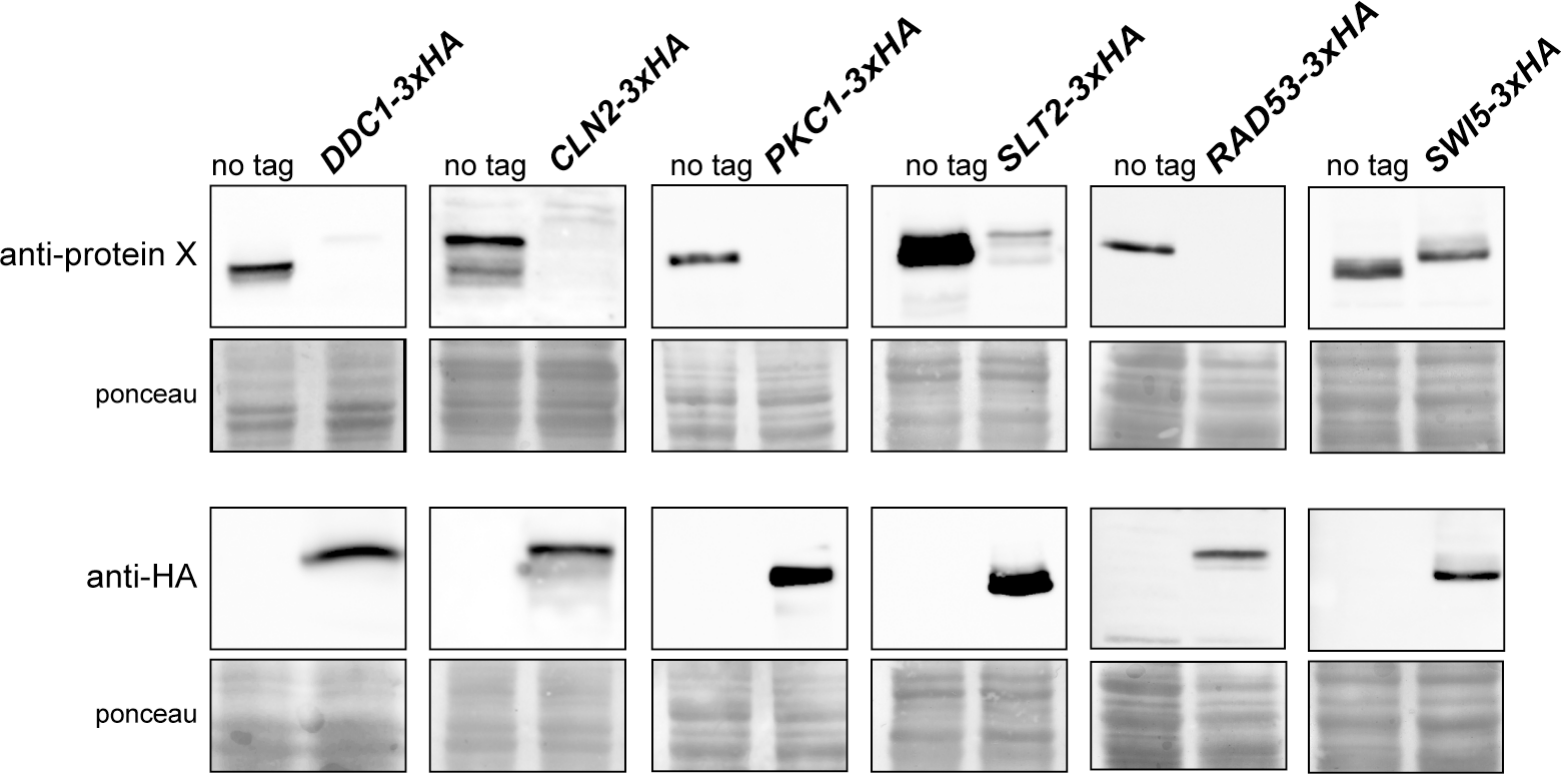
Analysis of the cellular content of different 3xHA tagged proteins. Cells of the wild-type (W303-1a), *DDC1-3xHA* (JCY1701), *CLN2-3xHA* (JCY1357), *PKC1-3xHA* (JCY2033), *SLT2-3xHA* (JCY411), *RAD53-3xHA* (JCY1905) and *SWI5-3xHA* (JCY487) strains were grown in YPD. Ddc1, Cln2, Pkc1, Slt2, Rad53 and Swi5 protein level in cell extracts was determined by western blot using specific anti-Ddc1, anti-Cln2, anti-Pkc1, anti-Slt2, anti-Rad53, anti-Swi5 or anti-HA antibodies. The ponceau staining of the membrane is shown as a loading control.

Small tags such as influenza virus hemagglutinin (HA) epitope are commonly used in laboratories to easily detect proteins with commercial antibodies. In our laboratory, we had tagged a large number of proteins at the C-terminus with the small peptide 3xHA using the transforming module described by Longtine *et al*. [13]. No problem was apparent when an anti-HA antibody was used. However, when an anti-Ddc1 antibody was used, we observed by western analysis an important reduction in the detected protein amount of C-terminal 3xHA tagged compared to non-labelled Ddc1 (Fig 1).

To further explore the relevance of this observation, we examined the influence of the C-terminal 3xHA tag in other five proteins: Cln2, Pkc1, Slt2, Rad53 and Swi5. As shown in Fig 1, western analysis with an anti-HA antibody confirmed the tagging of all proteins. Interestingly, as observed for Ddc1, a clear decrease in the detected amount of 3xHA-tagged Cln2, Pkc1, Slt2, Rad53 and in a lesser extent Swi5 proteins compared to their respective wild-type protein was revealed by the use of specific antibodies. The fact that this effect was detected in all six different proteins investigated, and that the same results were observed using a different yeast background (results not shown), strongly suggests that the observed reduction in protein level after C-terminal 3xHA tagging is not an artefact caused by altered antigen-antibody recognition but a genuine effect in protein level caused by the tagging.

### Protein level depends on the tagging introduced

**Fig 2:**
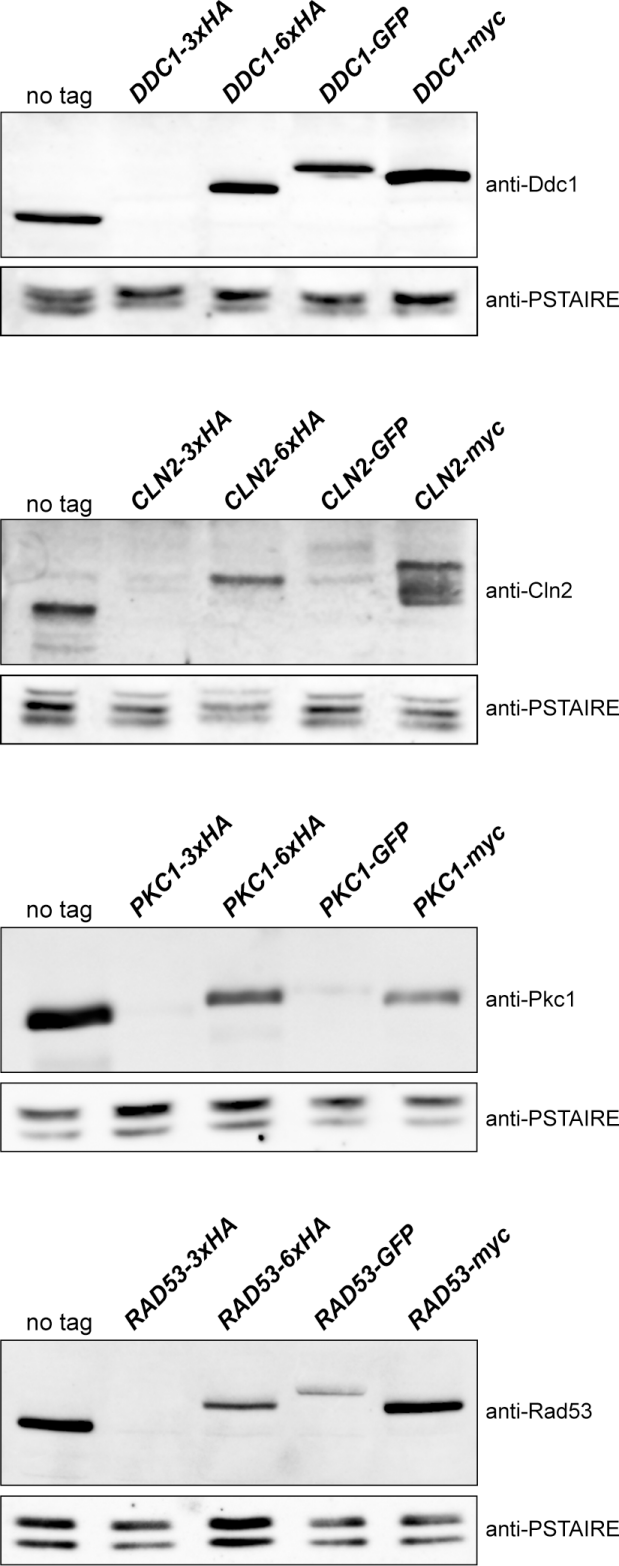
Analysis of the cellular content of proteins labelled with different tags. Cells of the wild-type (W303-1a), *DDC1-3xHA* (JCY1701), *DDC1-6xHA* (JCY1825), *DDC1-GFP* (JCY1661), *DDC1-myc* (JCY1887), *CLN2-3xHA* (JCY1357), *CLN2-6XHA* (JCY1830), *CLN2-GFP* (JCY1888), *CLN2-myc* (JCY1890), *PKC1-3xHA* (JCY2033), *PKC1-6XHA* (1891), *PKC1-GFP* (JCY1511), *PKC1-myc* (JCY1916), *RAD53-3xHA* (JCY1905), *RAD53-6XHA* (JCY1901), *RAD53-GFP* (JCY1903) and *RAD53-myc* (JCY1907) strains were grown in YPD. Protein level was determined by western blot using anti-Ddc1, anti-Cln2, anti-Pkc1 and anti-Rad53 antibodies. Cdc28 recognized by the anti-PSTAIRE antibody is shown as a loading control.

To investigate whether the decrease in protein level was caused by the structure of the transforming modules described in Longtine *et al*. [13] or by the addition of an HA epitope, we introduced different tags at the C-terminus of four of the six proteins. Concretely, Ddc1, Cln2, Pkc1 and Rad53 were tagged with GFP or myc using the Longtine *et al*. [13] modules and with HA using a different cassette containing 6 copies of the HA epitope (6xHA). The effect of the different taggings on protein level was investigated with the use of specific antibodies against the four proteins (Fig 2). On the contrary to that observed with the 3xHA tag, tagging with a different 6xHA cassette barely affected protein level. This result rules out that the reduction in protein level was due to HA sequences. Notably also, no effect at all (Ddc1, Cln2, Rad53) or a very mild effect (Pkc1) was observed in the case of myc-tagged proteins. In the case of GFP tagging, different responses were observed depending on the protein, response ranging from no effect in the case of Ddc1, a mild effect in Rad53 to a more important effect in the case of Cln2 and specially Pkc1. In short, these observations discard a general problem caused by the structure of transforming modules described in Longtine *et al*. [13], the problem being restricted to some singular cases, in particular to the use of the 3xHA cassette.

### Protein stability is affected when a 3xHA tagging is used

**Fig 3:**
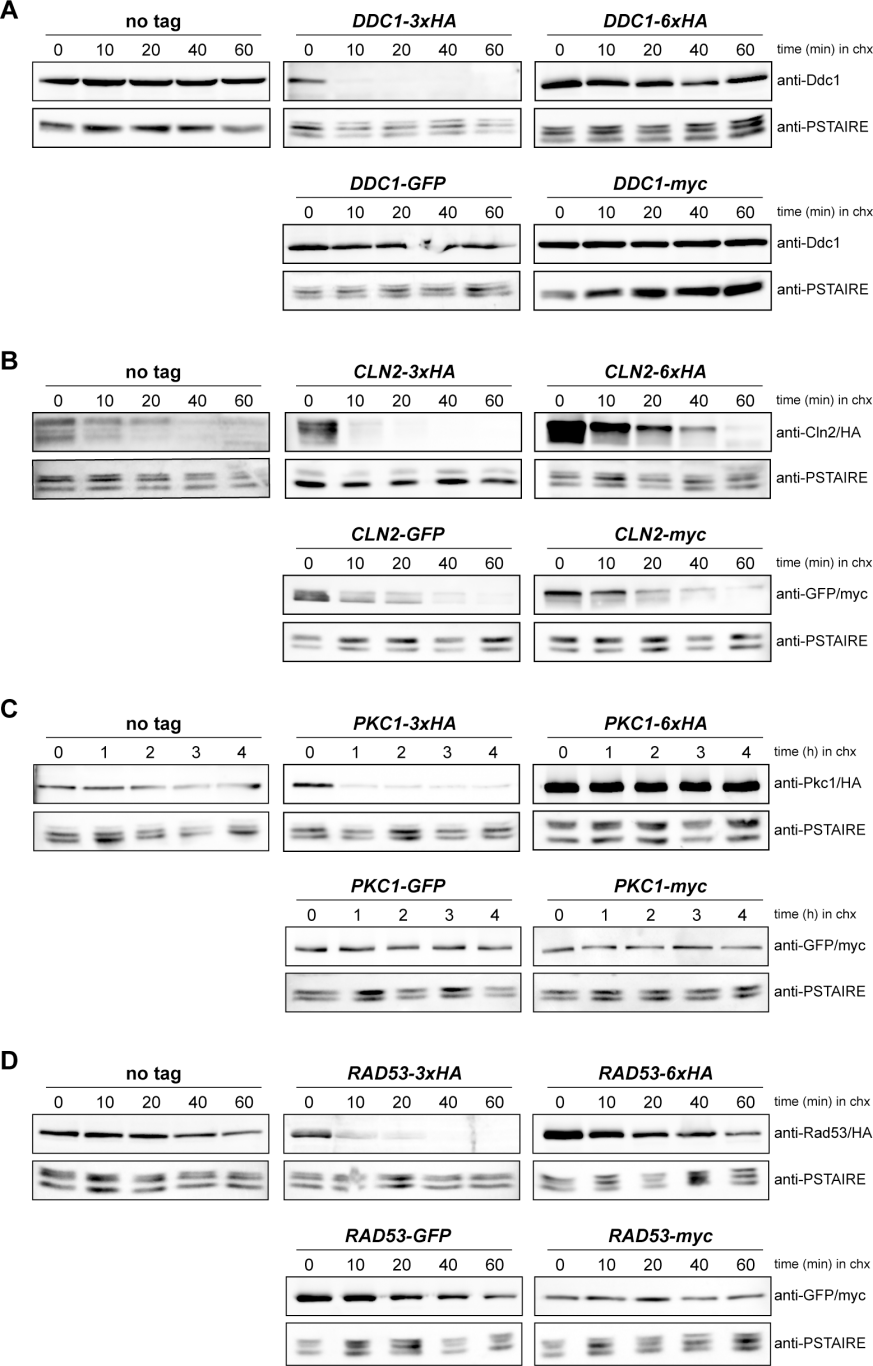
Analysis of the stability of proteins labelled with different tags. (A) Exponentially growing cultures of the wild-type (W303-1a), *DDC1-3xHA* (JCY1701), *DDC1-6xHA* (JCY1825), *DDC1-GFP* (JCY1661) and *DDC1-myc* (JCY1887) strains were incubated in the presence of 100 μg/mL cycloheximide. Ddc1 protein level was analysed at the indicated times after the addition of cycloheximide by western blot. Cdc28 recognized by the anti-PSTAIRE antibody is shown as a loading control. (B) Cln2 protein stability was analysed in wild-type (W303-1a), *CLN2-3xHA* (JCY1357), *CLN2-6XHA* (JCY1830), *CLN2-GFP* (JCY1888) and *CLN2-myc* (JCY1890) cells as described in A. (C) Pkc1 protein stability was analysed in wild-type (W303-1a), *PKC1-3xHA* (JCY2033), *PKC1-6XHA* (1891), *PKC1-GFP* (JCY1511) and *PKC1-myc* (JCY1916) cells as described in A. (D) Rad53 protein stability was analysed in wild-type (W303-1a), *RAD53-3xHA* (JCY1905), *RAD53-6XHA* (JCY1901), *RAD53-GFP* (JCY1903) and *RAD53-myc* (JCY1907) as described in A. Protein level was determined by western blot using anti-Ddc1, anti-Cln2, anti-Pkc1, anti-Rad53, anti-HA, anti-GFP or anti-myc antibodies as indicated. Cdc28 recognized by the anti-PSTAIRE antibody is shown as a loading control.

We wondered whether the decrease in protein levels was due to changes in protein stability as a consequence of the tagging. To investigate this possibility, translational shut-off experiments were carried out with proteins tagged with 3xHA, 6xHA, GFP and myc. We tested the stable proteins Ddc1, Pkc1 and Rad53 as well as the unstable Cln2 cyclin (Fig 3). In all cases, no significant differences in protein stability were observed when comparing 6xHA, GFP and myc tagged proteins to the wild-type protein. However, tagging with the 3xHA cassette caused a dramatic unstabilization of the protein in all cases.

The higher protein instability of the 3xHA-tagged proteins could certainly explain the reduced amount of protein detected in western blot analysis and confirm that this is a genuine effect of the tagging. It has been described that HA epitope contains a cryptic caspase-cleavage site [22], but our results with 6xHA tagging rules out that this could be the cause of the increased degradation rate. It has to be remarked that other transformation modules (GFP, myc) described in Longtine *et al*. [13] do not affect protein stability. In the case of some GFP tagged proteins that showed an apparent reduction in protein amount in western blot analysis, it could be speculated that tagging might affect the synthesis of the protein at any step or, alternatively, the big GFP tag could interfere with protein recognition by the specific antibody.

In short, we conclude that the 3xHA-tagging module causes, at least in the four proteins investigated, an important increase in protein instability leading to a reduced cellular protein content.

### Cells expressing 3xHA tagged proteins showed a reduced functional activity

**Fig 4:**
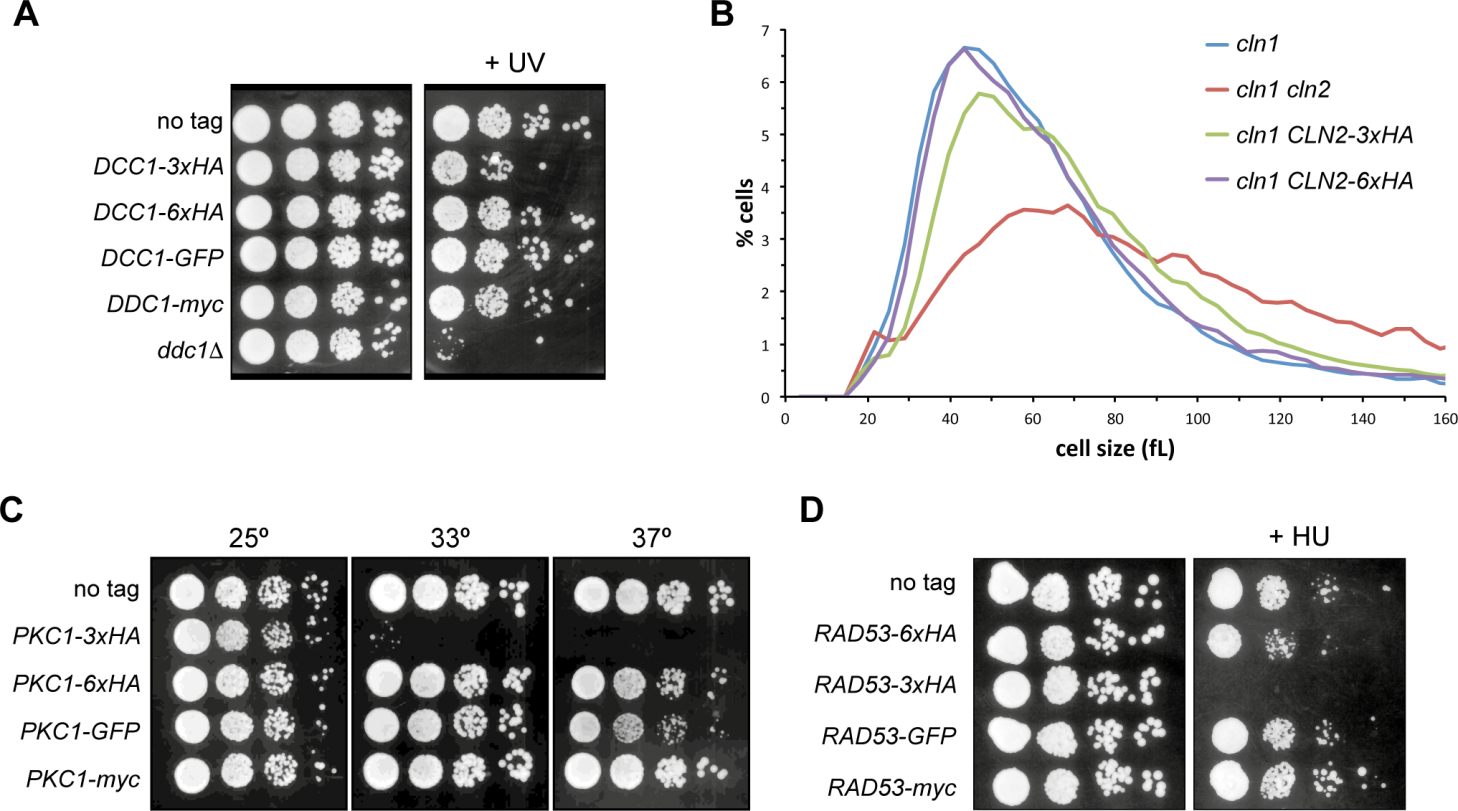
Functional analysis of proteins labelled with different tags. (A) 10-fold serial dilutions from exponentially growing cultures of wild-type (W303-1a), *DDC1-3xHA* (JCY1701), *DDC1-6xHA* (JCY1825), *DDC1-GFP* (JCY1661) and *DDC1-myc* (JCY1887) strains were spotted onto YPD medium and exposed to UV radiation (40 J/m^2^). Plates were incubated at 25°C for 4 days. (B) Cell size distribution in exponentially growing cultures of *cln1* (JCY275), *cln1 cln2* (JCY847), *cln1 CLN2-3xHA* (JCY929) and *cln1 CLN2-6XHA* (JCY1960) in complete SD medium. (C) 10-fold serial dilutions from exponentially growing cultures of wild-type (W303-1a), *PKC1-3xHA* (JCY2033), *PKC1-6XHA* (1891), *PKC1-GFP* (JCY1511) and *PKC1-myc* (JCY1916) were spotted onto YPD medium. Plates were incubated at 25°C, 33°C or 37°C for 3 days. (D) 10-fold serial dilutions from exponentially growing cultures of wild-type (W303-1a), *RAD53-6xHA* (JCY1901), *RAD53-3XHA* (JCY1905), *RAD53-GFP* (JCY1903) and *RAD53-myc* (JCY1907) were spotted onto YPD medium containing 200 mM hydroxyurea. Plates were incubated at 25°C for 3 days.

At this point, we analyzed whether the effect of the C-terminal 3xHA tagging could be reflected in protein functionality. Taking into account the role of each of the proteins analysed in this work, we investigated possible differences in the phenotype when yeast cells expressed the different labelled proteins. *DDC1* gene encodes for a subunit of the 9-1-1 complex involved in the DNA integrity checkpoint and its deletion causes sensitivity to different genotoxic agents, among them UV radiation [23]. In agreement with this, we observed that the *ddc1Δ* mutant cells were sensitive to non-lethal doses of UV irradiation (Fig 4A). Cells expressing GFP, myc or 6xHA tagged Ddc1 were able to grow to the same extent that wild-type cells after DNA damage. Nevertheless, the *DDC1-3xHA* strain showed an impaired cell growth, indicating that these cells are more sensitive to the UV radiation than those of wild-type or other Ddc1-tagged strains. This indicates a deficient Ddc1 function caused by the C-terminal 3xHA tagging, which is consistent with the reduced protein level.

Cln1 and Cln2 are cyclins involved in the regulation of the cell cycle, activating the CDK Cdc28 to promote the G1 to S phase transition [24, 25]. Cyclins Cln1 and Cln2 accumulate at the end of G1 at the Start transition and this is critical for the cell to enter in a new cycle at the right time. Inactivation of *CLN2* in a *cln1* background causes an important delay in the Start transition, detected as a substantial cell size increase [26]. Interestingly, whereas tagging of Cln2 with 6xHA in *cln1* cells had no effect on cell size, tagging with the 3xHA module caused a moderate but significant increase in cell size (Fig 4B). This result indicates that cells expressing the C-terminal 3xHA-tagged protein have a reduced Cln2 activity, which is consistent with the reduced protein level.

*PKC1* is an essential gene that encodes for a serine/threonine kinase involved in the maintenance of cell wall integrity [27], DNA integrity [28] and other cellular processes. The Pkc1 pathway is activated in response to a variety of stresses including heat shock stress [27]. Because of that, we carried out a growth assay at 33°C to test the functionality of the differentially tagged Pkc1 protein. As shown in Fig 4C, cells expressing 6xHA, myc and GFP tagged Pkc1 grew as well as wild-type cells at 33°C. In the case of *PKC1-GFP*, growth was slightly affected at 37°C, which is consistent with a reduced amount of protein detected in western blot analysis. Importantly, cells expressing 3xHA tagged Pkc1 were unable to grow at 33°C. This result indicates that C-terminal tagging of Pkc1 with the 3xHA module produces a reduced Pkc1 activity in the cell, which is consistent with the reduced protein level.

We also tested Rad53 functionality. Rad53 is the main effector kinase of the DNA integrity checkpoint and mediates most of the response in budding yeast cells [29]. Because of that, *RAD53* is an essential gene and defects in Rad53 activation cause hypersensitivity to genotoxic stress [30]. We analysed the growth of Rad53-labelled strains in the presence of hydroxyurea (HU), a replicative-stress inducing agent. As it can be seen in Fig 4D, in contrast to what is observed in wild-type, *RAD53-6xHA*, *RAD53-GFP* and *RAD53-myc* cells, the *RAD53-3xHA* cells could not grow in the presence of HU. This indicates that cells expressing Rad53 tagged at C-terminus with the 3xHA tag manifest a reduced Rad53 activity, which is consistent with the observed reduced protein level.

In summary, in all four cases investigated the C-terminal tagging with the 3xHA module causes a drastic unstabilization of the protein that leads to reduced cellular level and, hence, to a decreased activity of the protein in the cell.

### Protein stability depends on the amino acid spacer sequence between the protein and the 3xHA-tag

**Fig 5:**
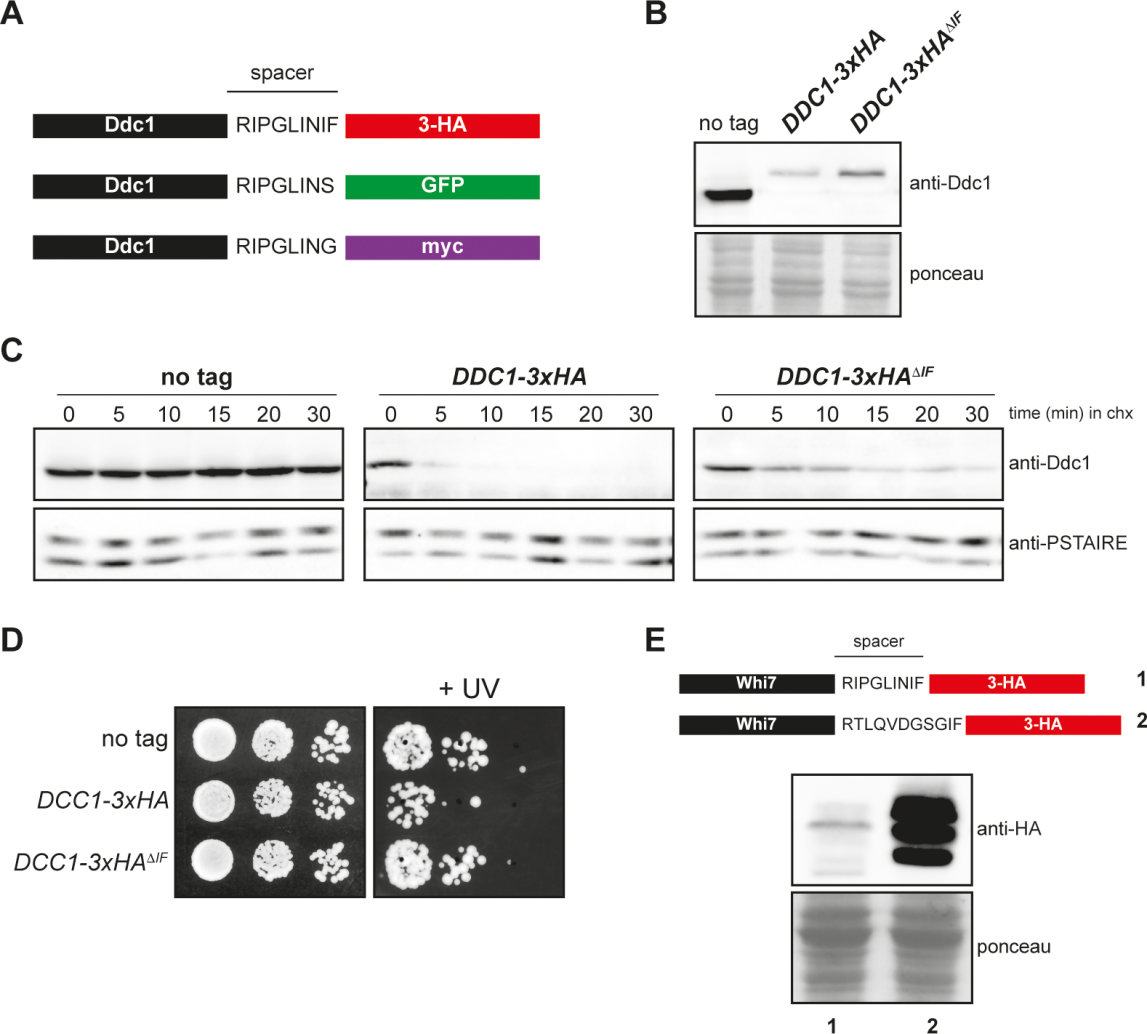
Analysis of the effect of the spacer sequence in 3xHA-tagging modules. (A) Schematic representation of Ddc1-3xHA, Ddc1-GFP and Ddc1-myc including the spacer sequence between the Ddc1 protein and the tag. (B) Cells of the wild-type (W303-1a), *DDC1-3xHA* (JCY1701) and *DDC1-3xHA*^*ΔIF*^ (JCY2063) strains were grown in YPD. Ddc1 protein level in cell extracts was determined by western blot using a specific anti-Ddc1 antibody. The ponceau staining of the membrane is shown as a loading control. (C) Exponentially growing cultures of the wild-type (W303-1a), *DDC1-3xHA* (JCY1701) and *DDC1-3xHA*^*ΔIF*^ (JCY2063) strains were incubated in the presence of 100 μg/mL cycloheximide. Ddc1 protein level was analysed at the indicated time after the addition of cycloheximide by western blot using an anti-Ddc1 antibody. Cdc28 recognized by the anti-PSTAIRE antibody is shown as a loading control. (D) 10-fold serial dilutions from exponentially growing cultures of wild-type (W303-1a), *DDC1-3xHA* (JCY1701) and *DDC1-3xHA*^*ΔIF*^ (JCY2063) strains were spotted onto YPD medium and exposed to UV radiation (40 J/m^2^). Plates were incubated at 25° for 4 days. (E) Whi7 was tagged with 3xHA using either the transforming modules described in Longtine *et al*. (JCY1544, lane 1) or an alternative cassette (JCY1728, lane 2). Whi7 protein level in cell extracts was determined by western blot using an anti-HA antibody. Note that Whi7 migrates in SDS-PAGE as multiple bands, which correspond to distinct phosphorylated states. The ponceau staining of the membrane is shown as a loading control.

Finally, in order to get insight into the unstabilization caused by the 3xHA tag used, we analysed the amino acid sequence of the spacer between the protein and the added tag in the transforming modules for C-terminal tagging described in Longtine *et al*. [13]. The spacers differ in the last amino acids, presenting the 3xHA cassette an Ile-Phe sequence absent in the other two cassettes (Fig 5A). To explore the influence of the spacer in the unstabilization problem described above, Ddc1 protein was labelled with a version of the 3xHA tag lacking the IF amino acids (3xHA^ΔIF^). As shown in Fig 5B, the elimination of these residues from the spacer caused an increase in the cellular content of Ddc1 when compared to the original 3xHA cassette, although the protein level is reduced in comparison to the non-tagged protein. Interestingly, and consistent with an increase in Ddc1 protein level, Ddc1-3xHA^ΔIF^ protein was significantly more stable than Ddc1-3xHA (Fig 5C) and Ddc1-3xHA^ΔIF^ carrying cells were less sensitive to non-lethal doses of UV irradiation than Ddc1-3xHA cells (Fig 5D). All these observations strongly suggest that the spacer between the protein and the 3xHA tag derived from these plasmids is involved in the reduction of protein level. Supporting this, we have observed a strong difference in the level of another yeast protein, Whi7, when tagged with the same 3xHA epitope but using a different cassette that differs in the spacer region (Fig 5E). This confirms that the specific spacer sequence present in the 3xHA-transforming module is the major determinant leading to the observed severe reduction in cellular protein content.

### Conclusions

In this work we have analysed different labelled proteins in budding yeast in order to characterize the influence of the tagging in protein level, stability and functionality. We have used the transformation modules (including 3xHA, myc and GFP tags) described in Longtine *et al*. [13], which are the most extensively used cassettes for tagging proteins in *S*. *cerevisiae*, and an independent 6xHA cassette.

Here we show that the use of these tagged proteins could be problematic because protein level could be severely affected. Surprisingly, on the contrary to most of previous published warnings about the convenience of using small tags [15], introduction of the small 3xHA-tag causes a strong decrease in protein level and a functional interference in all the analysed proteins. We analysed this effect in six different proteins for which specific antibodies were available in our laboratory, and it is remarkable that we observed an important reduction in the cellular content of the protein in all the investigated cases caused by a marked increase in protein instability. According to our results, better alternatives between the modules described in Longtine *et al*. [13] are the GFP or myc tags. The myc-tag is an optimal option because this tag renders normal protein expression, stability and functionality in the analysed proteins. GFP is also suitable because, although the protein signal detected can be affected in some cases, we have not observed a remarkable loss of functionality. Probably, although reduced, the remaining protein level may be enough to guarantee cell functions at least in the cases we have studied. Alternatively, it cannot be discarded that an impaired detection by the primary antibody against the protein could explain the reduced signal in GFP tagged proteins. In the case that an HA tag is desired to be used, we highly recommend alternative HA modules as the 6xHA used here, or other 3xHA modules with a different amino acid sequence in its spacer. Other authors have proposed to use small self-structured epitopes for innocuous protein tagging (inntags) to minimize the risk of functional and structural interference in protein taggings [17].

A final warning derived from our work is the necessity to be more careful when testing functionality of tagged proteins. It is noteworthy that loss of functionality of the labelled proteins could be difficult to detect because the strains phenotype is not affected in normal conditions. For instance, Pkc1 and Rad53 are essential proteins and the viability of *PKC1-3xHA* and *RAD53-3xHA* strains, with growth rates indistinguishable from wild-type strain, could mislead the researcher to consider these tagged proteins as fully functional, when they are not, as observed when more stringent growth conditions are tested. Similarly, *CLN2-3xHA* cells have the same cell size of wild-type cells suggesting that Cln2-3xHA is functional, but when combined with a *cln1* mutation, it is revealed that Cln2 activity is reduced after 3xHA tagging. Thus, based on positive detection of tagged proteins by western blot analysis and initial functional testing, many tagged proteins can be misused as normal proteins. Definitively, it is required extreme caution, more than initially thought, when working with tagged proteins.
